# Clinical Characteristics, Progression of Disease Within 24 Months (POD24), and Real-World Outcomes of Follicular Lymphoma in Saudi Arabia: A Two-Centre Retrospective Analysis

**DOI:** 10.3390/cancers18203311

**Published:** 2026-10-14

**Authors:** Mubarak Al-Mansour, Abdulrahman Salah Alharbi, Hadi Abdulhadi Sirdar, Aws Aljohani, Mohammed Abdu Gharwa, Hashim Khalid Alsendi, Samar Bernawi, Syed Sameer Aga, Manal A. Alsaggaf

**Affiliations:** 1 Adult Medical Oncology, Princess Noorah Oncology Centre, King Abdulaziz Medical City (KAMC), Ministry of National Guard Health Affairs-Western Region (MNGHA-WR), Jeddah 21451, Saudi Arabia; 2 Department of Basic Medical Sciences, College of Medicine, King Saud Bin Abdulaziz University for Health Sciences (KSAU-HS), King Abdullah International Medical Research Centre (KAIMRC), Ministry of National Guard Health Affairs-Western Region (MNGHA-WR), Jeddah 21423, Saudi Arabiaagas@ksau-hs.edu.sa (S.S.A.); 3 Oncology Centre, King Abdullah Medical City, Makkah 24246, Saudi Arabia; 4 College of Medicine, Umm Al-Qura University, Makkah 21955, Saudi Arabia

**Keywords:** follicular lymphoma, Saudi Arabia, POD24, early progression, bendamustine, rituximab, overall survival, progression-free survival, relapse

## Abstract

Background: Real-world evidence describing the presentation, management, and outcomes of follicular lymphoma (FL) in Saudi Arabia remains limited, and the frequency and prognostic weight of progression of disease within 24 months of first-line therapy (POD24) have not been reported in this population. This study evaluated the clinicopathological characteristics, treatment patterns, POD24 incidence, and survival outcomes of patients with FL treated at two Saudi tertiary centres. Methods: This two-centre retrospective chart review included adults with histologically confirmed FL diagnosed between November 1999 and May 2025. Overall survival (OS) and first-line progression-free survival (PFS) were measured from the initiation of first-line treatment and estimated using the Kaplan–Meier method. POD24 was defined as progression, relapse, or death from any cause within 24 months of first-line initiation among patients evaluable for that endpoint. Outcomes were additionally examined by calendar era of diagnosis. All group comparisons were unadjusted and exploratory. Results: A total of 139 patients were included. The mean age at diagnosis was 54.7 years (range, 18–91), 61.9% were male, 79.1% had Ann Arbor stage III–IV disease, and 51.1% had an FLIPI score ≥ 3. Bendamustine plus rituximab was the most frequent first-line regimen (43.9%), followed by R-CHOP (30.2%). The overall response rate was 77.0% in the whole cohort and 82.4% among the 125 patients who received response-evaluable systemic first-line therapy. After a median follow-up of 71.0 months, 5-year OS was 92.5% (95% CI, 84.8–96.4) and 5-year PFS was 66.1% (95% CI, 54.9–75.0); the corresponding medians were 210.0 and 114.6 months but were estimated with few patients at risk and should be interpreted cautiously. Among 97 patients evaluable for POD24, the event occurred in 24 (24.7%; 95% CI, 17.2–34.2), and the incidence was stable across calendar eras (23.4% before 2018 versus 24.5% thereafter; *p* = 1.000). Primary refractory disease accounted for 14 of the 24 events (58.3%). POD24 was associated with poorer ECOG performance status (odds ratio [OR] 11.08; 95% CI, 3.85–31.86), older age (OR 5.27; 95% CI, 2.00–13.89), and higher FLIPI score (OR 3.09; 95% CI, 1.13–8.42). In the 24-month landmark analysis, OS was significantly shorter after POD24 (log-rank *p* = 0.008), and both documented histological transformations occurred in the POD24 group. Conclusions: Patients with FL in this two-centre Saudi cohort frequently presented with advanced-stage and high-risk disease, yet contemporary anti-CD20-based treatment was associated with favourable long-term survival. Approximately one quarter of evaluable patients experienced POD24, predominantly as primary refractory disease, and this event identified a subgroup with materially inferior subsequent survival. POD24 may help identify patients who warrant closer surveillance and consideration for clinical trials or novel therapies.

## Simple Summary

Follicular lymphoma is a slow-growing blood cancer that usually responds well to treatment, but a minority of patients progress early and do considerably worse. Doctors identify this group using a marker called POD24—disease progression within 24 months of starting first treatment. POD24 has never been measured in Saudi Arabia. We reviewed 139 patients treated at two Saudi hospitals. Most did well, with 92.5% alive five years after starting treatment. However, about one in four patients evaluable for this marker progressed early, and their survival was substantially worse. Most of these early events happened during initial treatment rather than as later relapses, and the only two patients with documented transformation to an aggressive lymphoma were in this group. Recognising these patients early may help doctors consider closer monitoring and newer treatment options.

## 1. Introduction

Follicular lymphoma (FL) is an indolent B-cell non-Hodgkin lymphoma (NHL) arising from germinal centre B cells, which form characteristic follicular structures within lymphoid tissues [1]. It is the second most prevalent NHL subtype, accounting for approximately 20–30% of cases, with an annual incidence of 1.6 to 3.5 per 100,000 population [2,3]. The fifth edition of the World Health Organization Classification of Haematolymphoid Tumors (WHO-HAEM5) recognizes classic FL alongside distinct entities, such as in situ follicular B-cell neoplasm, pediatric-type FL, and duodenal-type FL, and treats histological grading of classic FL as optional [4]. Most cases harbor the t(14;18)(q32;q21) translocation, driving BCL2 overexpression and suppression of apoptosis [1]. Approximately 80% of patients present with advanced-stage disease (III–IV) [5]. FL generally follows a chronic, relapsing–remitting course, with a median overall survival (OS) of 10 to 20 years [6]; transformation to an aggressive lymphoma such as diffuse large B-cell lymphoma (DLBCL) occurs at a rate of 2–3% annually and is followed by a median OS of approximately 4.5 years [7].

Despite this generally favorable natural history, a clinically distinct subgroup experiences progression of disease within 24 months of first-line therapy (POD24). POD24 has been consistently identified as one of the most powerful early clinical determinants of subsequent mortality in FL and is increasingly used to select patients for intensified or investigational treatment strategies [8,9]. Its reported frequency ranges from approximately 15% to 25% depending on the treatment received and the analytic definition applied, yet no estimate has been published for a Saudi or wider Gulf population.

While FL is a recognized subtype of NHL globally, data from Saudi Arabia remain limited. Analyses from 2011 indicated that FL accounted for approximately 20.3% of NHL cases among Saudi adults (27 of 744 cases) [10]. A comprehensive two-decade national analysis of lymphoma incidence in Saudi Arabia documented a younger median age at presentation and a higher proportion of advanced-stage disease than in Western cohorts [11]. Hospital-based data from 2008 to 2012 indicated that approximately 44% of NHL cases presented at advanced stages [12]. That national analysis characterized incidence patterns but could not address treatment selection, response, or survival—the dimensions that determine outcome in routine practice. Real-world regional data describing how patients with FL are actually treated, how they respond, and how they fare over time are therefore needed to complement incidence-based surveillance. This two-center retrospective analysis describes the clinical characteristics, treatment patterns, and outcomes of patients with FL in Saudi Arabia and provides the first regional estimate of POD24 incidence together with its clinical correlates, subgroup composition, and prognostic impact.

## 2. Materials and Methods

### 2.1. Ethical Approval

This study was conducted in accordance with the principles of the Declaration of Helsinki, and the protocol was approved by the Institutional Review Boards (IRBs) of all participating centers [IRB/0749/23: 26 March 2023]. Given its retrospective design, the IRB waived the requirement for informed consent. All data were de-identified before analysis.

### 2.2. Study Design and Population

This two-center retrospective chart review used de-identified data from adult patients (aged ≥18 years at diagnosis) with FL, extracted from the electronic medical records of Princess Noorah Oncology Center, Jeddah, and King Abdullah Medical City, Makkah, Saudi Arabia. Consecutive patients aged 18 years or older with a diagnosis of FL established between November 1999 and May 2025 were eligible for inclusion. Histological diagnoses of FL were accepted, as documented in the institutional pathology reports issued at the time of diagnosis; archival material was not re-reviewed, and a central haematopathology review was not performed. Histological grade was recorded as assigned by the reporting pathologist according to the classification in use at that time, and the criteria for FL diagnosis and grading remained substantively unchanged across the accrual period. There were no restrictions regarding FL grade or treatment modality. Patients with a concurrent active malignancy other than non-melanoma skin cancer, as well as those who received their initial treatment at another institution, were excluded. The administrative data cut-off for follow-up was 1 August 2025, with a small number of records updated to the last documented contact through May 2026.

### 2.3. Data Collection and Definitions

Data were extracted using a standardized data collection form designed by the study investigators. Collected variables included baseline demographics (age, sex, and nationality), clinical presentation (B symptoms, Ann Arbor stage, Eastern Cooperative Oncology Group [ECOG] performance status, Follicular Lymphoma International Prognostic Index [FLIPI] score, bulky disease, and extranodal involvement), FL histological grade, and laboratory parameters. Treatment-related data included first-line regimen, use of maintenance rituximab, subsequent lines of therapy, radiotherapy, and stem cell transplantation (autologous or allogeneic). Treatment response was recorded as documented in the clinical record. For patients treated from 2014 onward, responses were assessed according to the Lugano 2014 response criteria; for patients treated before 2014, responses were recorded as documented under the criteria in use at the time, principally the International Working Group criteria. Responses were harmonized into four categories—complete response, partial response, stable disease, and progression—that are common to these criteria and were not retrospectively reassigned. Repeat biopsy at progression was performed at the treating physician’s discretion rather than systematically; documented histological transformation was recorded where available.

OS was defined as the time from initiation of first-line treatment to death from any cause; patients who were alive or lost to follow-up were censored at the date of last documented contact. First-line PFS was defined as the time from initiation of first-line treatment to documented progression, relapse, or death from any cause, whichever occurred first. Because exact progression dates were not uniformly recorded, PFS was reconstructed according to a pre-specified hierarchy: the date of first-line termination was used when the recorded best response was progression; the date of second-line initiation was used as a proxy when relapse or subsequent therapy was documented on or after first-line initiation; and the date of last follow-up was used as the date of death for patients recorded as deceased. When several candidate event dates were available, the earliest valid date was selected. Patients without a documented event were censored at the last valid clinical contact or response assessment. Patients for whom the start date or the event/censoring date was unavailable or internally inconsistent were classified as not evaluable for PFS. The median duration of follow-up was estimated using the reverse Kaplan–Meier method.

POD24 was defined as progression, relapse, or death from any cause within 24 months of the initiation of first-line treatment. Consistent with established practice, the POD24 analysis was restricted to patients evaluable for the endpoint, that is, those who either experienced an event within 24 months or who remained event-free with at least 24 months of follow-up; patients censored event-free before 24 months were uninformative for POD24 and were excluded from the denominator. Patients with POD24 were further classified as primary refractory (the best documented response to first-line therapy was progression), early relapse after complete or partial response, or progression after stable disease. Four sensitivity analyses were pre-specified: restriction to patients who received anti-CD20 antibody plus chemotherapy; a crude estimate using all PFS-evaluable patients irrespective of follow-up duration; a conservative estimate in which every documented but undated progression or relapse event was counted as a POD24 event; and restriction to events anchored to a documented progression or relapse, excluding proxy-dated events and deaths. A further sensitivity analysis excluded patients with grade 3B disease.

### 2.4. Statistical Analysis

Continuous variables were summarised as mean and range or median and interquartile range (IQR), and categorical variables as frequencies and percentages. The Kaplan–Meier method was used to estimate OS and PFS, and survival curves were compared using the log-rank test. The incidence of POD24 was reported with a Wilson score 95% confidence interval (CI). Baseline and treatment characteristics were compared between groups using the chi-square test or Fisher’s exact test for categorical variables, with a Monte Carlo estimate of the exact *p*-value (20,000 replicates) for tables larger than 2 × 2, and using the independent *t*-test (Welch correction) or the Mann–Whitney U test, as appropriate, for continuous variables. Effect sizes for the principal POD24 associations were expressed as odds ratios (ORs) with 95% CIs, calculated with the Haldane–Anscombe correction (0.5 added to each cell) for stability with sparse cells; CIs derived in this way may therefore differ marginally from the corresponding exact *p*-values at the significance boundary. Because patients with POD24 are by definition event-free survivors of the first 24 months only if they survive that period, post-POD24 survival was compared using a 24-month landmark analysis restricted to patients alive at the landmark, with survival time measured from that point. Outcomes were additionally examined by calendar era of diagnosis (1999–2012, 2013–2017 and 2018–2025 descriptively, with ≤2017 versus 2018–2025 used for formal comparison because of the small number of patients diagnosed before 2013). A two-sided *p*-value below 0.05 was considered statistically significant. No adjustment was made for multiple comparisons; all group comparisons were unadjusted and are reported as exploratory and hypothesis-generating rather than as tests of independent prediction. Cox proportional hazards regression was not undertaken because the number of observed events was low, which would have limited the statistical power and reliability of multivariable modeling. Analyses were performed in Python 3.11 using the lifelines (v0.30), SciPy (v1.15), and statsmodels (v0.14) libraries.

## 3. Results

### 3.1. Baseline Characteristics

**Figure 1:**
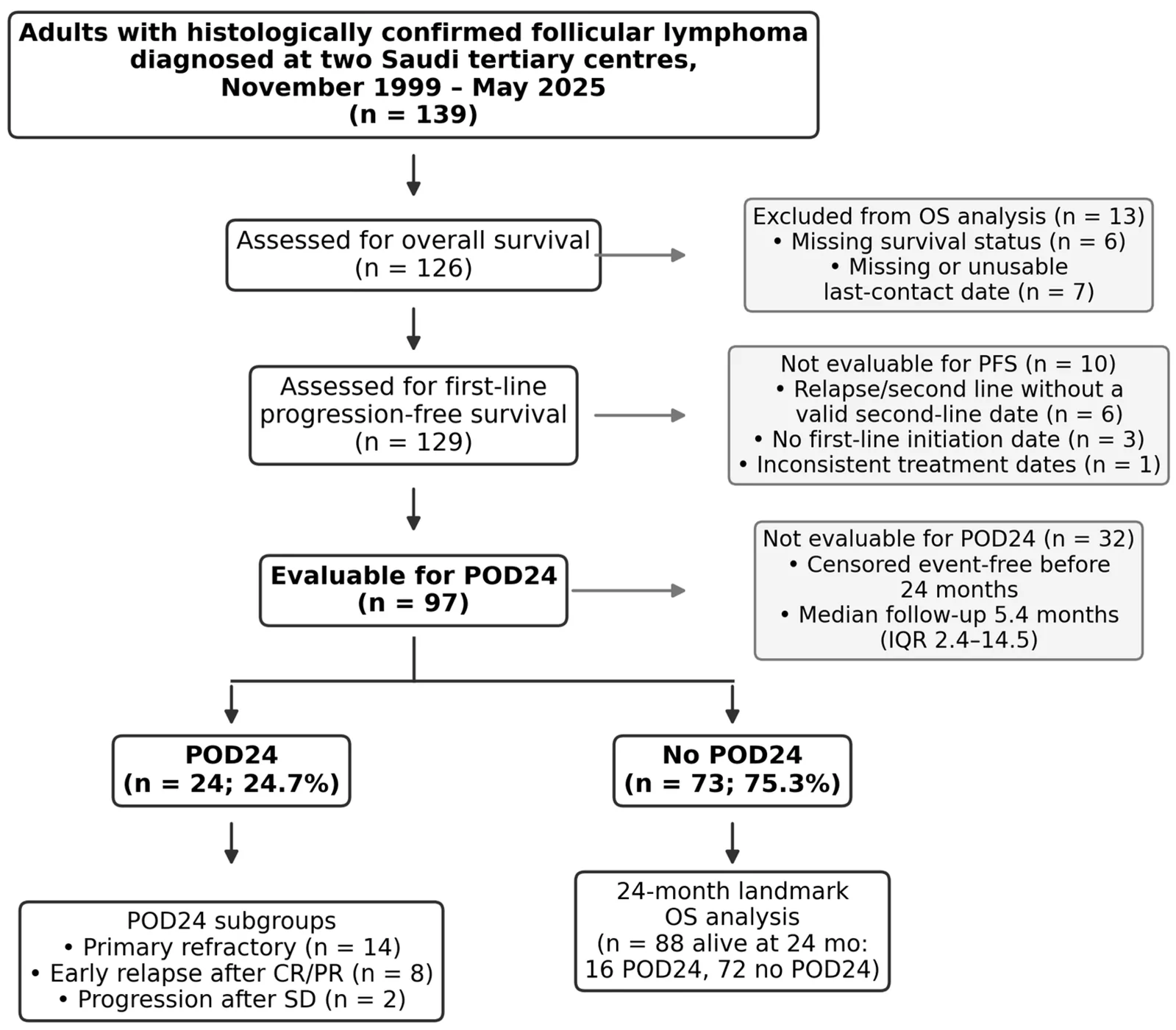
Flow of patients through the analysis populations. OS, overall survival; PFS, progression-free survival; POD24, progression of disease within 24 months of first-line therapy; CR, complete response; PR, partial response; SD, stable disease.

A total of 139 patients with follicular lymphoma were included in the analysis; the flow of patients through each analysis population is summarised in Figure 1. The mean age at diagnosis was 54.7 years (range, 18–91 years), and 61.9% of patients were male. Twelve patients (8.7%) were diagnosed between 1999 and 2012, 44 (31.9%) between 2013 and 2017, and 82 (59.4%) between 2018 and 2025. One POD24-evaluable patient had no recorded year of diagnosis and was therefore excluded from the era-stratified analysis, resulting in era totals of 96 patients. Advanced-stage disease (Ann Arbor stages III–IV) was present in 79.1% of patients, with stage IV the most frequent (54.7%). Histological grade 2 was the most common subtype (51.8%), followed by grade 1 (24.5%) and grade 3A (21.6%); grade 3B disease accounted for 2.2%. At diagnosis, elevated LDH was reported in 42.4% of patients, whereas low LDH was reported in 1.4%. Positive numeric beta-globulin measurements were available for 54 patients, with a mean value of 2.98 g/dL (range, 1.00–10.37 g/dL); elevated beta-globulin was documented in 13.7% of the total cohort. ECOG performance status was 0–1 in 55.4% of patients, 2 in 23.7%, and ≥3 in 20.9%. A FLIPI score ≥ 3 was recorded in 51.1% of patients. B symptoms were present in 30.9%, bone marrow involvement in 28.8%, extranodal involvement in 49.6%, and bulky disease in 33.8% (Table 1).

### 3.2. Treatment Patterns and Response

Bendamustine plus rituximab was the most frequently administered first-line regimen, used in 61 patients (43.9%), followed by R-CHOP in 42 (30.2%) and R-CVP in 13 (9.4%). Radiotherapy was the initial treatment strategy in 10 patients (7.2%), while watchful waiting and rituximab monotherapy were each used in 4 patients (2.9%). Among the 113 patients with an evaluable positive treatment interval, the mean duration of first-line treatment was 5.50 months (range, 1–85 months). The mean number of first-line treatment cycles was 5.61 (range, 1–8) among 122 patients with a valid positive cycle count. Maintenance rituximab was administered to 75 patients (54.0%). Following first-line therapy, complete response was achieved in 81 patients (58.3%) and partial response in 26 (18.7%), corresponding to an overall response rate of 77.0% (95% CI, 69.3–83.2) in the total cohort. Because the cohort-wide denominator includes patients managed with radiotherapy or watchful waiting, a restricted response rate was also calculated: among the 125 patients who received response-evaluable systemic first-line therapy, the overall response rate was 82.4% (95% CI, 74.8–88.1) and the complete response rate was 61.6%. Among the 117 patients treated with an anti-CD20 antibody plus chemotherapy, the overall response rate was 83.8% (95% CI, 76.0–89.4). Stable disease was observed in 16 patients (11.5%), progression in 14 (10.1%), and response status was not reported in 2 (1.4%; Table 2).

Second-line therapy was confirmed in 36 patients (25.9%). A specific second-line regimen was documented for 28 patients. Bendamustine plus rituximab was the most common second-line regimen (32.1%), followed by R-CHOP (21.4%), R-CVP (14.3%), R-ESHAP (7.1%), and obinutuzumab plus bendamustine (7.1%). The mean number of second-line treatment cycles was 4.69 ± 1.64 (range, 1–8) among 32 patients with a valid positive cycle count. Stem-cell transplantation was performed in 4 patients (2.9%; Table 2).

### 3.3. Overall and Progression-Free Survival

**Figure 2:**
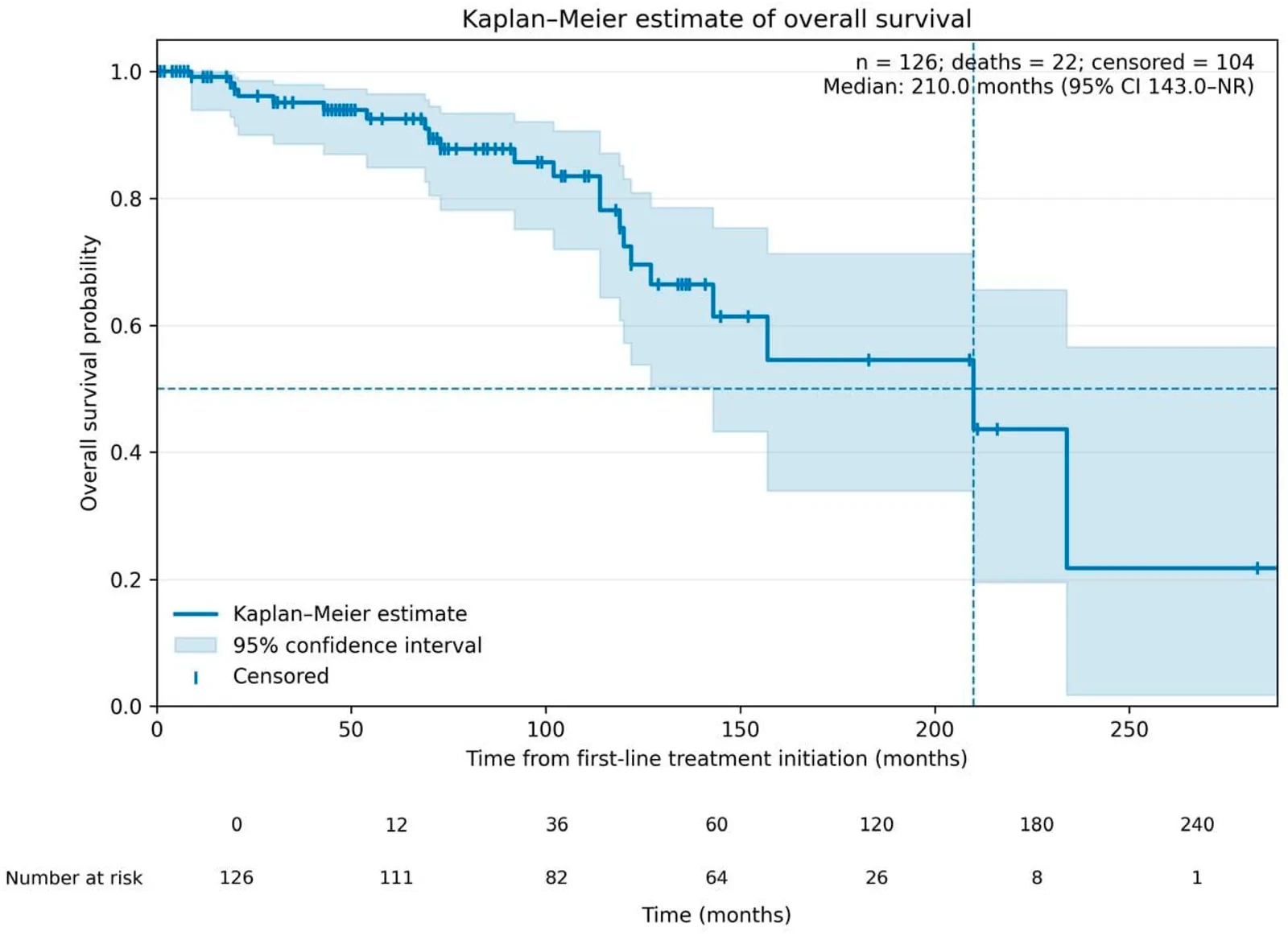
Kaplan–Meier estimate of overall survival in the overall cohort (n = 126), measured from the initiation of first-line treatment. Vertical marks denote censored observations; the shaded area denotes the 95% confidence interval. The number of patients at risk is shown below the *x*-axis.

**Figure 3:**
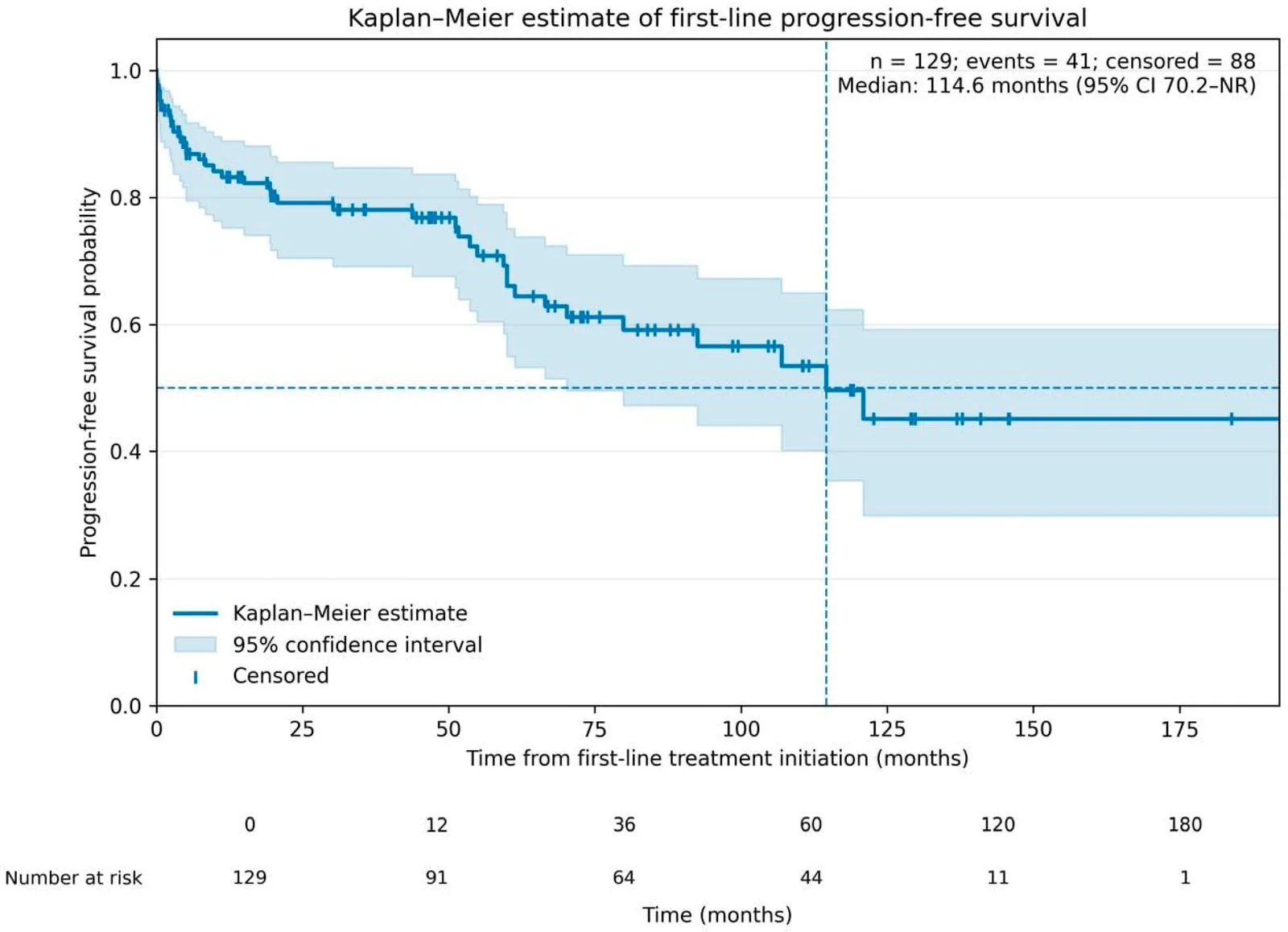
Kaplan–Meier estimate of first-line progression-free survival in the overall cohort (n = 129), measured from the initiation of first-line treatment. Vertical marks denote censored observations; the shaded area denotes the 95% confidence interval. The number of patients at risk is shown below the *x*-axis.

The median follow-up duration, estimated using the reverse Kaplan–Meier method, was 71.0 months (IQR, 30.0–129.0 months). OS was analyzed in 126 patients with evaluable survival status and follow-up time. During follow-up, 22 deaths were recorded (17.5%), while 104 patients were censored. The estimated OS probabilities at 12 months, 2 years, and 5 years were 99.1% (95% CI, 93.9–99.9), 96.1% (95% CI, 89.9–98.5), and 92.5% (95% CI, 84.8–96.4), respectively. First-line PFS was analyzed in 129 patients, among whom 41 progression or death events were observed (31.8%), while 88 patients were censored. The estimated PFS probabilities at 2 and 5 years were 79.1% (95% CI, 70.3–85.5) and 66.1% (95% CI, 54.9–75.0), respectively. The median OS was 210.0 months (95% CI, 143.0 months–not reached; Figure 2) and the median first-line PFS was 114.6 months (95% CI, 70.2 months–not reached; Figure 3); however, only five patients remained at risk at the estimated OS median and 14 at the PFS median, so these medians are unstable and the fixed-time estimates above should be regarded as the more reliable summary of outcome.

### 3.4. Outcomes by Era of Diagnosis

Outcomes were examined by calendar era of diagnosis to assess the influence of the prolonged accrual period. POD24 incidence was stable across eras: 1 of 10 evaluable patients diagnosed in 1999–2012 (10.0%; 95% CI, 1.8–40.4), 10 of 37 in 2013–2017 (27.0%; 95% CI, 15.4–43.0), and 12 of 49 in 2018–2025 (24.5%; 95% CI, 14.6–38.1; *p* = 0.530). In the two-era comparison, POD24 occurred in 11 of 47 patients diagnosed up to 2017 (23.4%) and 12 of 49 diagnosed from 2018 onward (24.5%; *p* = 1.000). First-line PFS was likewise similar (5-year PFS 64.6% versus 71.6%; log-rank *p* = 0.648). OS differed between eras (5-year OS 98.0% for patients diagnosed up to 2017 versus 85.3% for those diagnosed from 2018 onward; log-rank *p* = 0.001), despite a higher absolute number of deaths in the earlier group (13 of 51 versus 9 of 75). This pattern is most plausibly explained by survivor selection: patients diagnosed in the remote era could enter a retrospective chart review only if they remained in follow-up at the participating centers, so early deaths in that period are systematically under-ascertained. The era comparison of OS should therefore not be interpreted as evidence of deteriorating outcomes over time.

### 3.5. Incidence of Progression of Disease Within 24 Months (POD24)

Of the 129 patients evaluable for first-line PFS, 97 were evaluable for POD24; 32 patients were censored event-free before 24 months (median follow-up 5.4 months, IQR 2.4–14.5) and were therefore uninformative for this endpoint (Figure 1). POD24 occurred in 24 of 97 evaluable patients, corresponding to an incidence of 24.7% (95% CI, 17.2–34.2), while 73 patients (75.3%) remained free of progression, relapse, and death at 24 months. The qualifying events comprised progression documented at first-line response assessment in 14 patients, relapse in 4, an early second-line treatment proxy event in 4, and death in 2. The median time from first-line initiation to the POD24 event was 3.5 months (IQR, 0.7–8.8 months; range, 0.0–20.7 months); 21 of the 24 events (87.5%) occurred within the first 18 months, and only three occurred between 18 and 24 months. For comparison, the Kaplan–Meier estimate of the cumulative probability of progression or death by 24 months across all 129 PFS-evaluable patients was 20.9%.

Classified by the character of the qualifying event, 14 of the 24 patients with POD24 (58.3%) had primary refractory disease, 8 (33.3%) experienced early relapse after a complete (n = 3) or partial (n = 5) response, and 2 (8.3%) progressed after stable disease. The median time to event was 2.7 months in the primary refractory subgroup and 12.4 months in the early relapse subgroup, indicating that the POD24 population in this cohort was dominated by disease that never responded to induction rather than by classical early relapse. Histological transformation to DLBCL was documented in 2 patients (1.4% of the cohort), both of whom were in the POD24 group (2 of 24, 8.3%); no transformation was documented in the reference group. Because repeat biopsy was performed at the discretion of the treating physician rather than systematically, this proportion represents a lower bound, and the true transformation rate cannot be determined.

The estimate was stable across the pre-specified sensitivity analyses. Restricting the denominator to the 83 evaluable patients treated with an anti-CD20 antibody plus chemotherapy yielded a POD24 incidence of 20.5% (17 of 83; 95% CI, 13.2–30.4). A crude estimate including all 129 PFS-evaluable patients irrespective of follow-up duration yielded 18.6% (24 of 129; 95% CI, 12.8–26.2), which underestimates the true incidence because patients with insufficient follow-up cannot contribute events. A conservative estimate in which all seven documented but undated progression or relapse events were counted as POD24 events yielded 29.8% (31 of 104; 95% CI, 21.9–39.2). Restricting the analysis to the 18 events anchored to a documented progression or relapse, excluding proxy-dated events and deaths, yielded 19.8% (18 of 91; 95% CI, 12.9–29.1). Excluding the three patients with grade 3B disease yielded 23.4% (22 of 94; 95% CI, 16.0–32.9). The true incidence in this cohort, therefore, lies between approximately 20% and 30%.

To assess whether restriction to POD24-evaluable patients introduced selection bias, the 97 evaluable patients were compared with the 42 who were not evaluable (Table S1). The groups did not differ significantly in age (mean 54.6 versus 55.0 years; *p* = 0.884), sex (*p* = 0.845), advanced stage (81.4% versus 73.8%; *p* = 0.430), FLIPI ≥ 3 (54.6% versus 42.9%; *p* = 0.275), B symptoms (*p* = 0.844), bulky disease (*p* = 0.506), elevated LDH (*p* = 0.384), or receipt of an anti-CD20 antibody plus chemotherapy (86.6% versus 78.6%; *p* = 0.349). The groups differed by era of diagnosis (*p* = 0.010), with 78.6% of non-evaluable patients diagnosed from 2018 onward compared with 51.0% of evaluable patients, confirming that non-evaluability was driven principally by administrative censoring of recently diagnosed patients rather than by prognosis. ECOG distribution also differed (*p* = 0.049), but in the direction of fewer non-evaluable patients having an ECOG score ≥ 3 (11.9% versus 24.7%), the strongest correlate of POD24; had these patients been followed to 24 months, the expected POD24 incidence would therefore be no higher than that observed.

### 3.6. Clinical Characteristics Associated with POD24

Baseline characteristics of patients with and without POD24 are presented in Table 3. These comparisons are unadjusted and exploratory. Patients who experienced POD24 were older (mean 62.0 years, range 34–79, versus 52.3 years, range 18–91; *p* = 0.003), and 62.5% were aged 60 years or older compared with 23.3% of patients without POD24 (OR 5.27; 95% CI, 2.00–13.89) (Table 3). Performance status showed the strongest association: an ECOG score of ≥3 was recorded in 62.5% of patients with POD24 compared with 12.3% of those without POD24 (OR 11.08; 95% CI, 3.85–31.86), whereas an ECOG score of 0–1 was recorded in 25.0% and 67.1%, respectively (*p* < 0.001). The distribution of FLIPI scores also differed (*p* < 0.001), with a FLIPI score of 5 in 25.0% of the POD24 group compared with 2.7% of the reference group; a FLIPI score ≥ 3 was present in 75.0% versus 47.9% (OR 3.09; 95% CI, 1.13–8.42). B symptoms were more frequent in the POD24 group (50.0% versus 26.0%; OR 2.79; 95% CI, 1.09–7.15).

By contrast, Ann Arbor stage (OR 1.11; 95% CI, 0.34–3.58), histological grade, bone marrow involvement, extranodal involvement, bulky disease, sex, and LDH level did not differ significantly between the groups, although elevated LDH was numerically more common in the POD24 group (58.3% versus 41.1%; OR 1.97; 95% CI, 0.79–4.94). Patients with POD24 were less likely to have received an anti-CD20 antibody combined with chemotherapy (70.8% versus 90.4%; OR 3.12 for the absence of such therapy; 95% CI, 0.97–10.04). Maintenance rituximab was not analyzed as a baseline characteristic because it is administered only to patients who respond to and complete induction and is therefore conditioned on the absence of early progression; treating it as a baseline factor would be subject to guarantee-time bias. As expected from the endpoint definition, the response to first-line therapy differed markedly: complete response was documented in 12.5% of the POD24 group compared with 72.6% of the reference group, and progression at first response assessment accounted for 58.3% of the POD24 group (*p* < 0.001). Second-line therapy was subsequently administered to 18 of 24 patients with POD24 (75.0%) compared with 13 of 73 patients without POD24 (17.8%; *p* < 0.001). It should be noted that several of these variables are not independent of one another: FLIPI incorporates age, stage, haemoglobin, LDH, and nodal involvement, while performance status, FLIPI, and treatment selection are themselves interrelated, since impaired performance status influences both prognostic score and the choice of less intensive therapy. These features are therefore best interpreted as a single high-risk clinical phenotype rather than as separate, independent associations.

### 3.7. Survival After POD24

Patients who experienced POD24 had markedly inferior subsequent survival. Twelve of the 24 patients with POD24 died (50.0%) compared with 10 of the 73 patients without POD24 (13.7%). Measured from first-line initiation, median OS was 122.0 months in the POD24 group versus 234.0 months in the reference group (log-rank *p* < 0.001). Because this unlandmarked comparison is subject to guarantee-time bias, a 24-month landmark analysis was performed among the 88 patients who were alive at 24 months (16 with and 72 without POD24). From the landmark, the median OS was 103.0 months in the POD24 group compared with 210.0 months in the reference group (log-rank *p* = 0.008). The corresponding OS estimates at 5 years from the landmark were 84.6% (95% CI, 51.2–95.9) and 92.9% (95% CI, 82.0–97.3), respectively, with progressive divergence of the curves thereafter. Within the POD24 group, deaths occurred in 6 of 14 patients with primary refractory disease (42.9%) and 6 of 8 patients with early relapse after CR or PR (75.0%); in the landmark analysis, median OS from the landmark was 98.0 months and 103.0 months, respectively (log-rank *p* = 0.416). With only 14 and 8 patients in these subgroups, and with patients who died before 24 months necessarily excluded from the landmark, this comparison is descriptive, and no inference about the relative prognosis of the two subgroups is drawn. By definition, first-line PFS was substantially shorter in the POD24 group (median 3.5 months) than in the reference group, in which the median was not reached and the estimated 5-year PFS was 83.5%.

### 3.8. Characteristics Associated with Relapse

Baseline characteristics were also compared between patients who experienced relapse after first-line treatment and those who did not (Table 4). ECOG performance status differed significantly between the groups (*p* = 0.006), with poorer performance status being more frequent among patients who relapsed. An ECOG score of 0 was recorded in 6.5% of the relapse group compared with 30.6% of the no-relapse group, whereas an ECOG score of 3–5 was recorded in 38.7% and 15.7%, respectively. The FLIPI risk score and B symptoms demonstrated borderline associations with relapse (*p* = 0.052 for each). A FLIPI score of 5 was present in 19.4% of patients who relapsed compared with 3.7% of those without relapse, while B symptoms were present in 45.2% and 26.9%, respectively. Maintenance rituximab use did not differ significantly between the groups (*p* = 0.766).

The comparison of first-line management with relapse was repeated after collapsing the eleven recorded strategies into four clinically meaningful categories, because several original categories contained only one patient and the overall test was correspondingly unstable. Relapse occurred in 27 of 118 patients treated with an anti-CD20 antibody plus chemotherapy (22.9%), 0 of 14 managed with radiotherapy or watchful waiting (0%), 2 of 4 treated with rituximab monotherapy (50.0%), and 2 of 3 who received other chemotherapy regimens (66.7%); the association remained statistically significant (*p* = 0.028) but was attenuated relative to the eleven-category comparison (*p* = 0.002). The signal was driven by the absence of relapse among patients managed expectantly or with radiotherapy and by the small rituximab monotherapy group, rather than by any difference between individual immunochemotherapy regimens. There was accordingly no evidence that the choice among anti-CD20-containing chemotherapy regimens influenced relapse, and the zero-relapse cell may itself partly reflect less intensive radiological surveillance among patients managed expectantly.

## 4. Discussion

This two-center retrospective study describes the presentation, treatment, and outcomes of follicular lymphoma in Saudi Arabia and provides the first regional estimate of POD24. After a median follow-up of 71.0 months, the 5-year OS and first-line PFS were 92.5% and 66.1%, respectively. POD24 occurred in 24.7% of evaluable patients, was stable across calendar eras, was dominated by primary refractory disease, and identified a subgroup with significantly inferior subsequent survival.

The predominance of advanced-stage disease was consistent with the recognized presentation of FL, which is frequently disseminated before diagnosis because of its indolent clinical course [13]. The distribution was also concordant with Saudi and international treatment frameworks, in which stages III–IV are considered advanced disease and treatment initiation is determined primarily by tumor burden, symptoms, organ compromise, cytopenias, and performance status rather than stage alone [10]. The mean age of 54.7 years was younger than that reported in several Western population-based cohorts; for example, the median age was 62 years in a recent SEER cohort [14]. This difference most plausibly reflects referral patterns and institutional case selection rather than distinct disease biology and is discussed further in the limitations section.

Bendamustine plus rituximab was the most frequent first-line systemic regimen (43.9%), consistent with the Saudi Lymphoma Group guideline, which identifies it as a preferred option for advanced-stage FL requiring treatment while R-CHOP remains an accepted alternative [10]; this pattern aligns with the randomized StiL and BRIGHT trials [15,16].

The cohort-wide overall response rate of 77.0% was lower than the rates reported in the StiL and BRIGHT trials [15,16], but the denominators are not comparable: the cohort-wide figure includes patients managed with observation and radiotherapy, whereas the trials enrolled selected treatment-eligible populations with protocol-defined, centrally reviewed response assessment. Restricted to the 125 patients who received response-evaluable systemic first-line therapy, the overall response rate was 82.4%, and among those treated with an anti-CD20 antibody plus chemotherapy, it was 83.8%—figures that are directly comparable with trial experience, and that should be regarded as the clinically meaningful estimates for this cohort.

The estimated 5-year OS of 92.5% was consistent with modern rituximab-era series, in which 5-year OS has ranged from 90.8% to 92.7% [17]. The 5-year PFS of 66.1% was likewise within the range reported after contemporary immunochemotherapy and maintenance, with a projected 10-year PFS of 51.1% in PRIMA [18] and a 7-year PFS of 63.4% with obinutuzumab-based immunochemotherapy in GALLIUM [19].

These comparisons indicate that outcomes in the present cohort were broadly consistent with international experience in the anti-CD20 era, although cross-study comparisons remain limited by differences in eligibility, stage distribution, treatment selection, maintenance exposure, follow-up, and endpoint ascertainment. Because only five patients remained at risk at the estimated OS median and 14 at the PFS median, the median estimates are unstable, and the fixed-time 5-year estimates are the more reliable basis for comparison.

The identification of patients at risk of early progression remains clinically important. The POD24 incidence of 24.7% observed here was closely aligned with the 23% reported among evaluable patients in the population-based Danish national cohort [8] and lay within the range described in the National LymphoCare Study, which first established early progression after R-CHOP as a marker of increased mortality [9]. Restriction to patients treated with an anti-CD20 antibody plus chemotherapy produced a lower estimate of 20.5%, closer to the 15–20% typically reported in trial-derived immunochemotherapy populations, consistent with the observation that patients managed with radiotherapy or single-agent rituximab experienced early progression more frequently. Importantly, POD24 incidence did not differ across calendar eras (23.4% before 2018 versus 24.5% thereafter), indicating that the estimate is not an artifact of the prolonged accrual period. The prognostic separation in the landmark analysis reinforces the external evidence: median OS from the 24-month landmark was 103.0 months after POD24 versus 210.0 months without POD24 (*p* = 0.008), qualitatively similar to the Danish 10-year OS estimates of 66% versus 86% [8]. The separation emerged only after several years of follow-up, with 5-year landmark OS estimates of 84.6% and 92.9%, suggesting that the excess mortality associated with POD24 in this population was expressed over the medium rather than the immediate term.

The clinical correlates of POD24 identified here were consistent with previously reported associations. Poorer ECOG performance status showed the strongest association (OR 11.08; 95% CI, 3.85–31.86), followed by older age (OR 5.27) and higher FLIPI score (OR 3.09), mirroring the Danish analysis in which performance status ≥ 2 remained associated with progression within 24 months after multivariable adjustment (HR, 1.75; *p* = 0.008), alongside high FLIPI score, advanced stage, elevated LDH, and elevated β2-microglobulin [8]. The Ann Arbor stage, by contrast, showed essentially no association in the present cohort (OR 1.11; 95% CI, 0.34–3.58). Because only 24 events were available and no multivariable model could be fitted, these associations are unadjusted and should not be interpreted as independent predictors. A distinctive feature of this cohort was the composition of the POD24 group: 58.3% had primary refractory disease with a median time to event of only 2.7 months, rather than classical early relapse after response. Both documented histological transformations to DLBCL also occurred within the POD24 group. Patients whose disease never responds to induction are unlikely to be salvaged by anti-CD20 retreatment alone, and bispecific antibodies, CAR T-cell therapy, or clinical trial enrolment merit consideration at the earliest opportunity.

Relapse was documented in 22.3% of the cohort. ECOG performance status differed significantly between patients with and without relapse, with an ECOG score of 3–5 present in 38.7% and 15.7%, respectively—clinically plausible, because impaired performance status may reflect greater disease burden, comorbidity, or reduced tolerance of optimal treatment. The FLIPI score and B symptoms showed borderline associations (*p* = 0.052 for each). FLIPI remains an established prognostic instrument, but its principal function is risk stratification for survival rather than individual prediction of relapse, and its discriminatory performance is incomplete in patients treated with modern immunochemotherapy [20]. First-line management was associated with relapse after regrouping into four categories (*p* = 0.028), but the association reflected management strategy—the absence of relapse among patients managed expectantly or with radiotherapy and the high relapse proportion in the four patients given rituximab monotherapy—rather than any difference between individual immunochemotherapy regimens.

Several limitations should be considered. First, the retrospective design introduced susceptibility to incomplete documentation, variable follow-up intensity, and non-standardized response assessment; beta-globulin status was not reported in 63.3% of patients. Second, first-line PFS and POD24 were reconstructed from available clinical data. No POD24 event had an independently recorded radiologic progression date; every event date was anchored to a documented clinical event—the end of first-line therapy, the start of second-line therapy, or death. Misclassification across the 24-month threshold is nevertheless unlikely, because 21 of 24 events occurred within 18 months, and the sensitivity analysis restricted to events anchored to a documented progression or relapse gave a similar estimate (19.8%). Seven patients had a documented but undated progression or relapse and could not be assigned a POD24 status, and 32 were censored event-free before 24 months. Third, histological transformation could not be systematically ascertained because repeat biopsy was performed at physician discretion; the documented rate of 1.4% is a lower bound. Fourth, only 22 deaths and 41 analyzable PFS events were observed, precluding multivariable modeling, so all reported associations are unadjusted, uncorrected for multiple comparisons, and exploratory; cause of death was unavailable, preventing lymphoma-specific or competing-risk analysis. Fifth, the cohort accrued over more than two decades. Although POD24 and PFS were stable across eras, the era comparison of OS is confounded by survivor selection, and central pathology review was not performed, which is most relevant to grade 3A/3B assignment and to undetected transformation. Sixth, and most importantly for interpretation, the cohort was drawn from two tertiary referral centers and is not population-based. The relatively young mean age (54.7 years) and the high proportion of high-risk patients (FLIPI ≥ 3 in 51.1%) are characteristic of referral enrichment and differ from the national incidence patterns described by Basudan et al. [11]. These findings should therefore not be taken as a national reference for the incidence or case mix of FL in Saudi Arabia; the treatment patterns, response rates, and survival outcomes remain informative, but generalization to the wider Saudi population is not supported. Finally, molecular features, PET-derived parameters, treatment toxicity, quality of life, and patient-reported outcomes were unavailable.

The study nevertheless had several strengths. A relatively large Saudi FL cohort was assembled across two centres, detailed clinicopathological and treatment data were available, and follow-up was sufficiently prolonged to estimate medium- and long-term survival and to permit a genuine 24-month landmark analysis. The transparent reconstruction of OS, PFS, and POD24, the pre-specified sensitivity analyses bounding the POD24 estimate, the era-stratified analysis, and the retention of missing data rather than unsubstantiated imputation together enabled explicit characterization of both the findings and the limits of the available evidence.

## 5. Conclusions

This two-center Saudi cohort demonstrated that follicular lymphoma commonly presented with advanced-stage and high-risk clinical features, while contemporary anti-CD20-based treatment was associated with favorable long-term survival, with a 5-year OS of 92.5% and a 5-year PFS of 66.1%. Approximately one quarter of evaluable patients experienced progression of disease within 24 months of first-line therapy; this proportion was stable across calendar eras, was dominated by primary refractory disease, and identified a subgroup with materially inferior subsequent survival. Associations with older age, poorer performance status, and higher FLIPI score were unadjusted and exploratory. POD24 may help identify patients who warrant closer surveillance and consideration for clinical trials or novel therapies, although this retrospective analysis cannot demonstrate that treatment intensification based on POD24 improves outcome. Future Saudi registries should capture exact dates of progression, relapse, transformation, retreatment, and death so that POD24 can be determined prospectively and confirmed in population-based cohorts.

## Figures and Tables

**Table 1:** Baseline Clinical and Pathological Characteristics of the Study Cohort (N = 139).

Characteristic	No. (%) or Mean ± SD (Range)
**Age at diagnosis, y, mean (range) (n = 139)**	54.7 (18–91)
**Era of diagnosis**	
**1999–2012**	12 (8.7)
**2013–2017**	44 (31.9)
**2018–2025**	82 (59.4)
**Not recorded**	1 (0.7)
***Sex***	
**Male**	86 (61.9)
**Female**	53 (38.1)
***Ann Arbor stage at diagnosis***	
**I**	11 (7.9)
**II**	18 (12.9)
**III**	34 (24.5)
**IV**	76 (54.7)
***Histologic grade***	
**I**	34 (24.5)
**II**	72 (51.8)
**IIIa**	30 (21.6)
**IIIb**	3 (2.2)
**Haemoglobin at diagnosis, g/dL (n = 138)**	12.50 ± 2.37 (1.0–17.8)
**WBC at diagnosis, ×10^9^/L (n = 138)**	10.96 ± 26.19 (0.56–216.00)
**Platelets at diagnosis, ×10^9^/L (n = 136)**	250.96 ± 140.77 (1–906)
**LDH at diagnosis, U/L (n = 136)**	261.22 ± 196.05 (1–2090)
***Level of LDH***	
**Low**	2 (1.4)
**Normal**	73 (52.5)
**High**	59 (42.4)
**Not reported**	5 (3.6)
**Beta globulin at diagnosis, g/dL (n = 54)**	2.98 ± 1.48 (1.00–10.37)
***Level of beta globulin***	
**Low**	3 (2.2)
**Normal**	29 (20.9)
**High**	19 (13.7)
**Not reported**	88 (63.3)
***ECOG performance status***	
**0**	35 (25.2)
**1**	42 (30.2)
**2**	33 (23.7)
**3**	22 (15.8)
**4**	6 (4.3)
**5**	1 (0.7)
***FLIPI risk score***	
**0**	11 (7.9)
**1**	21 (15.1)
**2**	36 (25.9)
**3**	33 (23.7)
**4**	28 (20.1)
**5**	10 (7.2)
**B symptoms present**	43 (30.9)
***Bone marrow involvement***	
**No**	95 (68.3)
**Yes**	40 (28.8)
**Not done**	4 (2.9)
***Extranodal involvement***	
**No**	70 (50.4)
**Yes**	69 (49.6)
***Bulky disease***	
**No**	92 (66.2)
**Yes**	47 (33.8)

Abbreviations: ECOG, Eastern Cooperative Oncology Group; FLIPI, Follicular Lymphoma International Prognostic Index; LDH, lactate dehydrogenase; WBC, white blood cell count. Zero laboratory values were treated as missing. Isolated positive values of 1 were retained because no additional missing-value code was specified.

**Table 2:** Treatment Patterns and Responses (N = 139).

Variable	No. (%) or Mean ± SD (Range)
***First-line treatment/management strategy***	
**Bendamustine + rituximab**	61 (43.9)
**R-CHOP**	42 (30.2)
**R-CVP**	13 (9.4)
**Radiotherapy**	10 (7.2)
**Watch and wait**	4 (2.9)
**Rituximab monotherapy**	4 (2.9)
**ABVD**	1 (0.7)
**AVD**	1 (0.7)
**FCR**	1 (0.7)
**RCD**	1 (0.7)
**Obinutuzumab + bendamustine**	1 (0.7)
**Duration of first-line treatment, mo (n = 113)**	5.50 ± 9.55 (1–85)
**Number of cycles, first line (n = 122)**	5.61 ± 1.35 (1–8)
***Response to first-line therapy***	
**Complete response**	81 (58.3)
**Partial response**	26 (18.7)
**Stable disease**	16 (11.5)
**Progression**	14 (10.1)
**Not reported**	2 (1.4)
***Second-line therapy received***	
**Yes**	36 (25.9)
**No**	47 (33.8)
**Not reported**	56 (40.3)
***Second-line regimen (n = 28)***	
**Bendamustine + rituximab**	9 (32.1)
**R-CHOP**	6 (21.4)
**R-CVP**	4 (14.3)
**R-ESHAP**	2 (7.1)
**Obinutuzumab + bendamustine**	2 (7.1)
**R-GDP**	1 (3.6)
**Bendamustine monotherapy**	1 (3.6)
**HD-MTX and IT**	1 (3.6)
**Radiotherapy**	1 (3.6)
**Polatuzumab + rituximab**	1 (3.6)
**Number of cycles, second line (n = 32)**	4.69 ± 1.64 (1–8)
***Stem cell transplantation***	
**Yes**	4 (2.9)
**No**	135 (97.1)

Abbreviations: ABVD, doxorubicin/bleomycin/vinblastine/dacarbazine; AVD, doxorubicin/vinblastine/dacarbazine; FCR, fludarabine/cyclophosphamide/rituximab; HD-MTX, high-dose methotrexate; IT, intrathecal therapy; Pola-R, polatuzumab plus rituximab; RCD, rituximab/cyclophosphamide/dexamethasone; R-CHOP, rituximab/cyclophosphamide/doxorubicin/vincristine/prednisone; R-CVP, rituximab/cyclophosphamide/vincristine/prednisone; R-ESHAP, rituximab/etoposide/methylprednisolone/cytarabine/cisplatin; R-GDP, rituximab/gemcitabine/dexamethasone/cisplatin. First-line treatment duration was calculated as completed calendar months and included positive intervals among treated patients; watch-and-wait records and zero-month intervals were excluded. Cycle summaries included positive numeric values only.

**Table 3:** Baseline and treatment characteristics according to POD24 status among patients evaluable for the 24-month endpoint (n = 97).

Characteristic	POD24 (n = 24)	No POD24 (n = 73)	*p*-Value	OR (95% CI)
**Age at diagnosis, y, mean (range)**	62.0 (34–79)	52.3 (18–91)	0.003	
**Age ≥ 60 years**	15 (62.5)	17 (23.3)		5.27 (2.00–13.89)
**Male sex**	15 (62.5)	44 (60.3)		—
**Ann Arbor stage III–IV**	20 (83.3)	59 (80.8)		1.11 (0.34–3.58)
***Histological grade***			0.195	
**Grade 1**	6 (25.0)	21 (28.8)		
**Grade 2**	8 (33.3)	35 (47.9)		
**Grade 3A**	8 (33.3)	16 (21.9)		
**Grade 3B**	2 (8.3)	1 (1.4)		
***ECOG performance status***			<0.001	
**0–1**	6 (25.0)	49 (67.1)		
**2**	3 (12.5)	15 (20.5)		
**≥3**	15 (62.5)	9 (12.3)		11.08 (3.85–31.86)
**FLIPI score ≥ 3**	18 (75.0)	35 (47.9)		3.09 (1.13–8.42)
**B symptoms present**	12 (50.0)	19 (26.0)		2.79 (1.09–7.15)
**Bone marrow involvement**	9 (39.1)	23 (31.5)		
**Extranodal involvement**	14 (58.3)	37 (50.7)		
**Bulky disease**	9 (37.5)	26 (35.6)		
**Elevated LDH**	14 (58.3)	30 (41.1)		1.97 (0.79–4.94)
**Anti-CD20 antibody plus chemotherapy**	17 (70.8)	66 (90.4)		3.12 (0.97–10.04) *
***Best response to first-line therapy***			<0.001	
**Complete response**	3 (12.5)	53 (72.6)		
**Partial response**	5 (20.8)	13 (17.8)		
**Stable disease**	2 (8.3)	7 (9.6)		
**Progression**	14 (58.3)	0 (0.0)		
**Received second-line therapy**	18 (75.0)	13 (17.8)	<0.001	

Data are presented as n (%) unless otherwise indicated. POD24 was defined as progression, relapse, or death from any cause within 24 months of the initiation of first-line treatment; the analysis was restricted to the 97 patients evaluable for this endpoint. Percentages are calculated within each column. All comparisons are unadjusted and exploratory, and no correction was applied for multiple comparisons. *P*-values were derived from the chi-square test or Fisher’s exact test for categorical variables (Monte Carlo estimation of the exact *p*-value for tables larger than 2 × 2) and from Welch’s *t*-test for continuous variables. Odds ratios were calculated with the Haldane–Anscombe correction (0.5 added to each cell) and may therefore differ marginally from the corresponding exact *p*-values at the significance boundary. Maintenance rituximab is not shown because it is administered only after successful induction and is therefore conditioned on the absence of early progression. * OR for the absence of anti-CD20 antibody plus chemotherapy. ECOG, Eastern Cooperative Oncology Group; FLIPI, Follicular Lymphoma International Prognostic Index; LDH, lactate dehydrogenase; OR, odds ratio; POD24, progression of disease within 24 months; SD, standard deviation.

**Table 4:** Comparison of Baseline Characteristics Between Patients With and Without Relapse After First-Line Treatment.

Characteristic	No Relapse (n = 108)	Relapse (n = 31)	*p*-Value
**Age at diagnosis, y, mean (range)**	54.8 (18–91)	54.4 (34–84)	0.912
**Sex**			0.621
**Female**	40 (37.0)	13 (41.9)	
**Male**	68 (63.0)	18 (58.1)	
**Ann Arbor stage at diagnosis**			0.481
**I**	10 (9.3)	1 (3.2)	
**II**	12 (11.1)	6 (19.4)	
**III**	26 (24.1)	8 (25.8)	
**IV**	60 (55.6)	16 (51.6)	
**Histologic grade**			0.598
**I**	28 (25.9)	6 (19.4)	
**II**	53 (49.1)	19 (61.3)	
**IIIa**	25 (23.1)	5 (16.1)	
**IIIb**	2 (1.9)	1 (3.2)	
**LDH level**			0.468
**Low**	1 (0.9)	1 (3.2)	
**Normal**	60 (55.6)	13 (41.9)	
**High**	43 (39.8)	16 (51.6)	
**Not reported**	4 (3.7)	1 (3.2)	
**Beta globulin level**			0.632
**Low**	2 (1.9)	1 (3.2)	
**Normal**	25 (23.1)	4 (12.9)	
**High**	14 (13.0)	5 (16.1)	
**Not reported**	67 (62.0)	21 (67.7)	
**ECOG performance status**			0.006
**0**	33 (30.6)	2 (6.5)	
**1**	35 (32.4)	7 (22.6)	
**2**	23 (21.3)	10 (32.3)	
**3**	14 (13.0)	8 (25.8)	
**4**	3 (2.8)	3 (9.7)	
**5**	0 (0.0)	1 (3.2)	
**FLIPI risk score**			0.052
**0**	8 (7.4)	3 (9.7)	
**1**	19 (17.6)	2 (6.5)	
**2**	29 (26.9)	7 (22.6)	
**3**	27 (25.0)	6 (19.4)	
**4**	21 (19.4)	7 (22.6)	
**5**	4 (3.7)	6 (19.4)	
**B symptoms**			0.052
**No**	79 (73.1)	17 (54.8)	
**Yes**	29 (26.9)	14 (45.2)	
**Bone marrow involvement**			0.874
**No**	75 (69.4)	20 (64.5)	
**Yes**	30 (27.8)	10 (32.3)	
**Not done**	3 (2.8)	1 (3.2)	
**Extranodal involvement**			0.803
**No**	55 (50.9)	15 (48.4)	
**Yes**	53 (49.1)	16 (51.6)	
**Bulky disease**			0.823
**No**	72 (66.7)	20 (64.5)	
**Yes**	36 (33.3)	11 (35.5)	
**First-line treatment/management strategy**			0.002
**Bendamustine + rituximab**	52 (48.1)	9 (29.0)	
**R-CHOP**	32 (29.6)	10 (32.3)	
**R-CVP**	5 (4.6)	8 (25.8)	
**Radiotherapy**	10 (9.3)	0 (0.0)	
**Watch and wait**	4 (3.7)	0 (0.0)	
**Rituximab monotherapy**	2 (1.9)	2 (6.5)	
**ABVD**	1 (0.9)	0 (0.0)	
**AVD**	0 (0.0)	1 (3.2)	
**FCR**	0 (0.0)	1 (3.2)	
**RCD**	1 (0.9)	0 (0.0)	
**Obinutuzumab + bendamustine**	1 (0.9)	0 (0.0)	
**Maintenance rituximab**			0.766
**No**	49 (45.4)	15 (48.4)	
**Yes**	59 (54.6)	16 (51.6)	

Data are presented as n (%) unless otherwise indicated. Percentages were calculated within each relapse group. The age comparison was performed using an independent-samples Student’s *t* test. Categorical comparisons were performed using Pearson chi-square tests without continuity correction, consistent with the original analysis. Sparse treatment categories should be interpreted cautiously. Age was unavailable for one patient in the relapse group; the age comparison therefore included 108 patients without relapse and 30 patients with relapse.

## Data Availability

The de-identified datasets generated and analysed during the current study are available from the corresponding author on reasonable request, subject to institutional and regulatory approval.

## Supplementary Materials

The following supporting information can be downloaded at https://www.mdpi.com/article/10.3390/cancers18203311/s1. Table S1. Comparison of baseline characteristics between patients evaluable and not evaluable for POD24.
