# Supplementary material for: Clinical Characteristics, Progression of Disease Within 24 Months (POD24), and Real-World Outcomes of Follicular Lymphoma in Saudi Arabia: A Two-Centre Retrospective Analysis
Source: Cancers (Basel). 2026 Oct 14;18(20):3311. doi: 10.3390/cancers18203311 (PMC13702530; doi:10.3390/cancers18203311)
Supplement: Supplementary file 1 [file cancers-18-03311-s001.zip › cancers-4551160-supplementary.pdf]

Table S1. Comparison of baseline characteristics between patients evaluable and not evaluable for POD24.

Characteristic	Category	Evaluable (n = 97)	Not evaluable (n = 42)	p value
**Age at diagnosis, years**	mean (range)	54.6 (18-91)	55.0 (27-86)	0.884
**Sex**	Female	38 (39.2)	15 (35.7)	0.845
**Sex**	Male	59 (60.8)	27 (64.3)	
**Ann Arbor III-IV**	No	18 (18.6)	11 (26.2)	0.430
**Ann Arbor III-IV**	Yes	79 (81.4)	31 (73.8)	
**Histological grade**	Grade 1	27 (27.8)	7 (16.7)	0.053
**Histological grade**	Grade 2	43 (44.3)	29 (69.0)	
**Histological grade**	Grade 3A	24 (24.7)	6 (14.3)	
**Histological grade**	Grade 3B	3 (3.1)	0 (0.0)	
**ECOG**	0-1	55 (56.7)	22 (52.4)	0.049
**ECOG**	2	18 (18.6)	15 (35.7)	
**ECOG**	>=3	24 (24.7)	5 (11.9)	
**FLIPI >=3**	No	44 (45.4)	24 (57.1)	0.275
**FLIPI >=3**	Yes	53 (54.6)	18 (42.9)	
**B symptoms**	Absent	66 (68.0)	30 (71.4)	0.844
**B symptoms**	Present	31 (32.0)	12 (28.6)	
**Bulky disease**	No	62 (63.9)	30 (71.4)	0.506
**Bulky disease**	Yes	35 (36.1)	12 (28.6)	
**Elevated LDH**	No	53 (54.6)	27 (64.3)	0.384
**Elevated LDH**	Yes	44 (45.4)	15 (35.7)	
**Anti-CD20 + chemo**	No	13 (13.4)	9 (21.4)	0.349
**Anti-CD20 + chemo**	Yes	84 (86.6)	33 (78.6)	
**Era of diagnosis**	1999-2012	10 (10.4)	2 (4.8)	0.010
**Era of diagnosis**	2013-2017	37 (38.5)	7 (16.7)	
**Era of diagnosis**	2018-2025	49 (51.0)	33 (78.6)	

Data are n (%) unless otherwise indicated. Comparisons used the chi-square or Fisher's exact test for categorical variables (Monte Carlo estimation for tables larger than 2 × 2) and Welch's t-test for age. ECOG, Eastern Cooperative Oncology Group; FLIPI, Follicular Lymphoma International Prognostic Index; LDH, lactate dehydrogenase; POD24, progression of disease within 24 months.
